# Microbe Profile: *Wigglesworthia glossinidia*: the tsetse fly’s significant other

**DOI:** 10.1099/mic.0.001242

**Published:** 2022-09-21

**Authors:** Brian L. Weiss, Rita V. M. Rio, Serap Aksoy

**Affiliations:** ^1^​ Department of Epidemiology of Microbial Diseases, Yale School of Public Health, New Haven, CT, USA; ^2^​ Department of Biology, Eberly College of Arts and Sciences, West Virginia University, Morgantown, WV, USA

**Keywords:** *Wigglesworthia*, tsetse fly, mutualist, endosymbiont, vitamin provisioning, immune enhancement

## Abstract

*
Wigglesworthia glossinidia* is an obligate, maternally transmitted endosymbiont of tsetse flies. The ancient association between these two organisms accounts for many of their unique physiological adaptations. Similar to other obligate mutualists, *Wigglesworthia *’s genome is dramatically reduced in size, yet it has retained the capacity to produce many B-vitamins that are found at inadequate quantities in the fly’s vertebrate blood-specific diet. These *Wigglesworthia *-derived B-vitamins play essential nutritional roles to maintain tsetse’s physiological homeostasis as well as that of other members of the fly’s microbiota. In addition to its nutritional role, *Wigglesworthia* contributes towards the development of tsetse’s immune system during the larval period. Tsetse produce amidases that degrade symbiotic peptidoglycans and prevent activation of antimicrobial responses that can damage *Wigglesworthia *. These amidases in turn exhibit antiparasitic activity and decrease tsetse’s ability to be colonized with parasitic trypanosomes, which reduce host fitness. Thus, the *Wigglesworthia* symbiosis represents a fine-tuned association in which both partners actively contribute towards achieving optimal fitness outcomes.

## Abbreviations

PGRP-LB, peptidoglycan recognition protein LB; SAM, S-adenosyl methionine.

## Taxonomy

Phylum: *Proteobacteria *; Class: *Gammaproteobacteria *; Order: *Enterobacterales *; Family: *Erwiniaceae *; Genus: *Wigglesworthia *; Species: *Wigglesworthia glossinidia *. The species name is used for this clade of tsetse fly symbionts with host species appended to designate strains. The *Wigglesworthia* type strain was described in 1995 within *Glossina morsitans morsitans* [1]. The name is in recognition of insect physiologist Sir Vincent Brian Wigglesworth F.R.S. (1899–1994), who described the *Wigglesworthia *–tsetse symbiosis in 1929.

## Properties

Tsetse’s obligate endosymbiont *Wigglesworthia* presents two membrane bilayers, is rod-shaped and is approximately 5 µm in length. *Wigglesworthia* reside freely within the cytoplasm of specialized epithelial cells, known as bacteriocytes, which collectively form the bacteriome organ that is attached to tsetse’s anterior midgut. This population of *Wigglesworthia* provide their tsetse host with essential nutrients, including B-vitamins, that are lacking in physiologically sufficient quantities in the fly’s strict vertebrate blood-specific diet. A second population of *Wigglesworthia* resides extracellularly within the accessory (milk) gland tubules of females. Vertical transmission of *Wigglesworthia* occurs via milk gland secretions that seed the bacterium to the developing intrauterine larva.

## Genome

The genomes of *Wigglesworthia glossinidia* ‘morsitans’ and ‘brevipalpis’ have been fully sequenced and annotated [2, 3]. These genomes demonstrate high gene conservation and chromosomal synteny and have a median size of 0.7 Mb, 623 proteins and 23.8 % G+C nucleotide content. The genome contains two rRNA operons with contiguity between rRNA genes. The small genome size reflects the relaxed evolutionary selection on less relevant genes in this specialized lifestyle and their subsequent loss, and higher levels of genetic drift due to population bottlenecks during transmission. The genome is polyploid within individual *Wigglesworthia* cells. Notably, *Wigglesworthia* genomes lack the *dnaA* gene and *oriC* region that encode the DNA replication initiation protein and the origin of replication, respectively, probably indicating alternative modes of regulation. The retention of flagellum synthesis capabilities may confer motility to extracellular *Wigglesworthia* during vertical transmission and aid in the colonization of larval bacteriomes. A small cryptic plasmid (~5 kb) is retained by *Wigglesworthia *, suggesting functional relevancy towards symbiont and/or tsetse biology.

## Phylogeny

Based on 16S rRNA gene sequences, the genus *Wigglesworthia* forms a distinct lineage in the Gamma subdivision of *Proteobacteria* and in congruence with tsetse species phylogeny. These parallel phylogenies support an evolutionary scenario of an initial *Wigglesworthia* infection prior to tsetse species radiation and subsequent co-diversification of symbiont and tsetse host [4].

## Key features and discoveries

*Wigglesworthia contributions towards tsetse nutrition: Wigglesworthia*’s extensive and intimate co-evolution with the tsetse fly is reflected by the bacterium’s crucial metabolic contributions to the maintenance of tsetse’s fitness as well as the fitness of the fly’s other microbial partners. For example, *Wigglesworthia* is intimately tied to tsetse’s reproductive success. Pregnant female tsetse utilize proline as a prominent haemolymph-borne energy source to fuel the production of milk that serves as the sole nutrient source for developing intrauterine larvae. Vertebrate blood does not contain sufficient quantities of proline, and as such, female flies recycle alanine, which is a byproduct of the tricarboxylic acid cycle, back into proline. This process is facilitated by the enzyme alanine-glyoxylate aminotransferase, which requires *Wigglesworthia *-derived vitamin B6 (pyridoxal phosphate) as a co-factor. When pregnant female tsetse flies are treated with antibiotics to remove their *Wigglesworthia *, milk production ceases and larval offspring perish [5]. *Wigglesworthia* also produce vitamin B9 (folate), and experimental inhibition of folate production (via blood meal supplementation with glyphosate) prolongs tsetse blood meal digestion and the development of intrauterine larval and free-living pupal stages. Additionally, newly emerged adults derived from glyphosate-treated mothers present significantly smaller wings than do their counterparts derived from wild-type mothers [5].

Tsetse is not the only benefactor of *Wigglesworthia-*produced vitamins. African trypanosomes, which are transmitted by tsetse and cause socioeconomically devastating human and animal African trypanosomiases, are also folate auxotrophs that, like their fly host, rely on *Wigglesworthia* to provide this vitamin. Accordingly, inhibition of *Wigglesworthia* folate production reduces tsetse’s competence as a vector of pathogenic African trypanosomes [6]. Many tsetse flies also house the facultative endosymbiont *Sodalis *. This bacterium lacks the genetic machinery to produce vitamin B1 (thiamine), which it relies on for proper growth. *Wigglesworthia* produces thiamine, and *Sodalis* probably scavenges this vitamin from its environment via the activity of a thiamine ABC transporter [5]. The *Wigglesworthia* metabolic contributions described above provide evidence of how the obligate bacterium enhances tsetse’s fitness as well as the fitness of members of the fly’s microbial community.

*Wigglesworthia contributions towards tsetse immunity*: In addition to providing essential vitamins to the tsetse holobiont, *Wigglesworthia* is intimately associated with the development and proper function of the fly’s immune system. Tsetse offspring that undergo their entire developmental programme in the absence of *Wigglesworthia* present several immunocompromised phenotypes during adulthood. For example, *Wigglesworthia *-free flies fail to produce a structurally complete peritrophic matrix, which is a chitinous sleeve that lines the fly’s midgut and serves as a barrier to establishment of trypanosome infection. This phenotype contributes to the establishment and progression of trypanosome infections in tsetse’s midgut [7]. *Wigglesworthia *-free tsetse adults also present a highly depleted population of sessile and circulating haemocytes. These immune cells phagocytose foreign invaders and produce melanin that encapsulates pathogens and forms clots at cuticular wound sites [8]. These findings indicate that *Wigglesworthia* plays a crucial role in tsetse’s competence as a vector of trypanosomes as well as the fly’s ability to recover from injuries.

*Wigglesworthia–tsetse dialogue*: Gene expression analysis of the bacteriome organ reveals a closely tailored dialogue between tsetse and *Wigglesworthia *. Bacteriocytes produce an abundant peptidoglycan recognition protein LB (PGRP-LB) that has amidase activity to degrade symbiotic peptidoglycan, which is a major elicitor of host antimicrobial immune responses that can otherwise damage the bacteria [9], and a multivitamin transporter (smvt) that probably aids in the dissemination of nutrients provisioned by *Wigglesworthia* [10]. The *Wigglesworthia *-responsive PGRP-LB also has anti-parasitic activity and decreases tsetse’s ability to be colonized with parasitic trypanosomes, which in infected insects reduce host fitness [9]. *Wigglesworthia* overproduces molecular chaperones (GroEL and its co-factor GroES) that help facilitate the correct folding of its protein products given the unusually high AT bias (88 % A+T) of its genome. This molecular dialogue was also reflected in the metabolomic analysis from bacteriome and haemolymph collected from normal and symbiont-cured female tsetse. The absence of *Wigglesworthia* disrupted multiple host metabolic pathways, including those of carbohydrates and amino acids, and affected downstream nucleotide biosynthesis and metabolism and biosynthesis of *S*-adenosyl methionine (SAM), an essential cofactor [10].

*Future opportunities:* The conspicuously intimate nature of tsetse’s relationship with *Wigglesworthia* has heretofore prevented the cultivation of this bacterium outside of the fly. This obstacle has impeded our ability to identify and characterize in more depth the specific *Wigglesworthia *-derived molecules that enhance tsetse fitness. However, the fact that *Wigglesworthia* resides extracellularly in tsetse milk provides promise that the bacterium can be grown *in vitro* in a biochemically similar medium. Doing so will facilitate the ability to interfere with *Wigglesworthia* gene expression as a means of identifying fecundity-enhancing and immunostimulatory molecules that may be targetable in novel control strategies aimed at reducing tsetse population size and trypanosome vector competence.

## Open questions

1. What are the *Wigglesworthia *-derived factors that contribute to tsetse immune system development, and how do these factors function in this capacity?
2. What are the mechanisms that facilitate metabolite exchange and regulate coordination between *Wigglesworthia *, its tsetse host and other members of the fly’s microbiota?
3. How can we develop a medium in which we can cultivate (and potentially genetically modify) *Wigglesworthia* outside of its tsetse host?
4. How can *Wigglesworthia* be experimentally exploited to reduce tsetse’s competence as a vector of pathogenic African trypanosomes?
